# Severe Neutropenia is Associated with Better Clinical Outcomes in Patients with Advanced Pancreatic Cancer Who Receive Modified FOLFIRINOX Therapy

**DOI:** 10.3390/cancers10110454

**Published:** 2018-11-16

**Authors:** Yunami Yamada, Hironori Fujii, Daichi Watanabe, Hiroko Kato-Hayashi, Koichi Ohata, Ryo Kobayashi, Takuma Ishihara, Shinya Uemura, Takuji Iwashita, Masahito Shimizu, Akio Suzuki

**Affiliations:** 1 Department of Pharmacy, Gifu University Hospital, Gifu 501-1194, Japan; y_yunami@gifu-u.ac.jp (Y.Y.); watayaku@gifu-u.ac.jp (D.W.); hhayashi@gifu-u.ac.jp (H.K.-H.); k_ohata@gifu-u.ac.jp (K.O.); ryo_k@gifu-u.ac.jp (R.K.); akio@gifu-u.ac.jp (A.S.); 2 Gifu University Hospital, Innovative and Clinical Research Promotion Center, Gifu University, Gifu 501-1194, Japan; tishiha@gifu-u.ac.jp; 3 First Department of Internal Medicine, Gifu University Hospital, Gifu 501-1194, Japan; ueshin550621@gmail.com (S.U.); takuji@w7.dion.ne.jp (Takuji Iwashita); shimim-gif@umin.ac.jp (M.S.)

**Keywords:** modified FOLFIRINOX, severe neutropenia, overall survival, overall response rate, time to treatment failure, advanced pancreatic cancer

## Abstract

While modified FOLFIRINOX therapy is effective for treating advanced pancreatic cancer, it frequently causes severe neutropenia. The present study investigated the effect of severe neutropenia on clinical outcomes in advanced pancreatic cancer patients who received modified FOLFIRINOX. The study subjects were 51 patients (30 males and 21 females) with advanced pancreatic cancer who received modified FOLFIRINOX (2h bolus injection of oxaliplatin at 85 mg/m^2^, 2 h bolus injection of L-leucovorin at 200 mg/m^2^, 90min bolus injection of irinotecan at 150 mg/m^2^, followed by continuous infusion of 5-fluorouracil for 46 h at 2400 mg/m^2^ without bolus 5-fluorouracil) during the period from January 2014 to May 2018. No patients had prior history of chemotherapy. Adverse events, including neutropenia, were graded according to the Common Terminology Criteria for Adverse Events, version 4.0. Median overall survival (OS) was the primary endpoint, while median time to treatment failure (TTF), overall response rate (ORR), and the incidence of other adverse events were secondary endpoints. Severe neutropenia (grade ≥3) occurred in 39 patients (76.4%), and Cox proportional hazard analysis identified high total bilirubin level as a significant risk factor. Median duration of OS was significantly longer in patients with severe neutropenia than in those without it (21.3 months versus 8.9 months, *p* = 0.020). Moreover, there was a significant correlation between OS and the grade of neutropenia (r = 0.306, *p* = 0.029). ORR tended to be higher, though not significantly, in patients with severe neutropenia. In contrast, the incidence rates of other adverse events were not different between the two groups. Severe neutropenia is an independent predictor of prognosis in advanced pancreatic cancer patients received modified FOLFIRINOX therapy.

## 1. Introduction

Pancreatic cancer has extremely poor prognosis and represents the fourth leading cause of cancer-related mortality in the world. Among the various malignancies ten-year (4.6% for males and 4.8% for females) and five-year (7.0% for males and 5.9% for females) survival rates in Japan are the worst for pancreatic cancer [1,2]. There are few effective therapies for pancreatic cancer, indicating the urgent need for development of more effective chemotherapies to improve the poor outcomes of this malignancy.

FOLFIRINOX, a combination chemotherapy of oxaliplatin, irinotecan, 5-fluorouracil (5-FU), and L-leucovorin, has been shown to significantly prolong survival and is currently a standard chemotherapy for advanced pancreatic cancer. Conroy et al. showed that FOLFIRINOX therapy exhibited clinical superiority over gemcitabine monotherapy with respect to overall survival (OS), progression-free survival (PFS), and tumor response rate (RR) in patients with metastatic pancreatic cancer [3]. However, grade 3–4 neutropenia occurs more frequently in patients treated with FOLFIRINOX than gemcitabine (45.7% versus 21.0%, *p* < 0.001). A high incidence of grade 3–4 neutropenia (77.8%) has also been observed in Japanese patients receiving FOLFIRINOX for metastatic pancreatic cancer [4].

To reduce the incidence of toxicities associated with FOLFIRINOX, the chemotherapy regimen has been modified by omission the bolus injection of 5-FU and/or reducing the dose of irinotecan without reducing the clinical response [5,6,7,8,9]. Modification of the FOLFIRINOX regimen (oxaliplatin 85 mg/m^2^, irinotecan 150 mg/m^2^, 5-FU infusion 2400 mg/m^2^ over 46 h, no bolus 5-FU) reduces the incidence of grade 3–4 neutropenia to 47.8% [7]. More importantly, a meta-analysis showed that the modified FOLFIRINOX regimen is as effective as the original, with a similar tumor response rate (32% versus 33%, *p* = 0.879), rate of 12-month survival (47% versus 50%, *p* = 0.38), rate of 6-month PFS rate (47% versus 53%, *p* = 0.38) and reduced frequency of grade 3–4 adverse events [10].

The occurrence of severe adverse events, including neutropenia [11,12], mucositis [13], neurotoxicity [14] and diarrhea [15], during cancer chemotherapy results in impaired patient quality of life and leads to the therapy interruption or dose reduction. The latter may lead to a reduction in the therapeutic effect due to the decrease in the relative dose intensity (RDI). For example, the incidence of grade 4 neutropenia is significantly higher, while median PFS and OS are significantly shorter in non-small cell lung cancer patients receiving irinotecan with UGT1A1*6 homozygous mutation compared to the heterozygous mutation or wild-type allele [16]. The incidence rates of diarrhea and neutropenia are significantly higher, while median OS tends to be shorter in metastatic colorectal cancer patients receiving irinotecan with UGT1A1*28 heterozygous or homozygous mutations compared to the wild-type allele [17].

In contrary, several investigators have shown that the occurrence myelosuppression such as neutropenia is a surrogate for better survival in patients receiving cancer chemotherapy. In patients with metastatic colorectal cancer who receive FOLFOX therapy, the incidences of mild (grade 1–2) and severe (grade 3–4) neutropenia are associated with improved survival [18]. In patients with advanced pancreatic cancer who received gemcitabine alone or in combination with other anticancer drugs, the median OS is significantly longer in patients with early onset of neutropenia than in those without neutropenia [19]. Comparison of the effect of gemcitabine monotherapy on survival among metastatic pancreatic patients with grade 3 neutropenia, those with grade 1–2 neutropenia and those without neutropenia also shows that the median survival time is prolonged with increasing grade of neutropenia [20]. Therefore, the incidence neutropenia may have different effects on the survival period, depending on the cancer type and chemotherapy regimen.

We investigated the relationship between the incidence of severe neutropenia and OS in patients with advanced pancreatic cancer who received modified FOLFIRINOX as first-line chemotherapy to determine whether or not the incidence of severe neutropenia causes favorable survival outcomes in these patients

## 2. Results

### 2.1. Patient Demographics

Among the 51 studied patients, 30 (58.8%) were male and 21 (41.2%) were female, and the median age was 64 years (Table 1). The prevalence of heterozygous for *UGT1A1* was 37.3% (Table 1), in which UGT1A1*6/*1 appeared in 19.6% and UGT1A1*28/*1 in 17.6%. At the start of chemotherapy, there was no significant difference in body surface area; body mass index; hemoglobin; the numbers of white blood cells, neutrophil, and platelet; or the number of patients with biliary stent or drainage between the two groups.

The overall incidence rate of severe neutropenia (grade ≥3) was 76.5% (39/51). Among the 39 patients, severe neutropenia predominantly occurred during the first cycle (82%, 32 patients), but also occurred during the second cycle (10%, four patients) and during the third cycle (8%, three patients). Demographics were compared between patients with and without severe neutropenia. Granulocyte-colony stimulating factor (G-CSF) was not used for primary prevention of neutropenia in any patients. However, 34 of the 39 patients who developed severe neutropenia were administered G-CSF for recovery from neutropenia. The RDI of irinotecan, oxaliplatin, and 5-FU was 0.73, 0.49, and 0.65 in patients with severe neutropenia, and 0.84, 0.74, and 0.83 in patients without severe neutropenia, respectively. Therefore, the RDI was significantly lower in patients with severe neutropenia than in those without severe neutropenia for irinotecan (*p* = 0.034), oxaliplatin (*p* < 0.001) and 5-FU (*p* < 0.001).

### 2.2. Comparison of the Efficacy of Modified FOLFIRINOX between Patients with and without Severe Neutropenia

**Figure 1:**
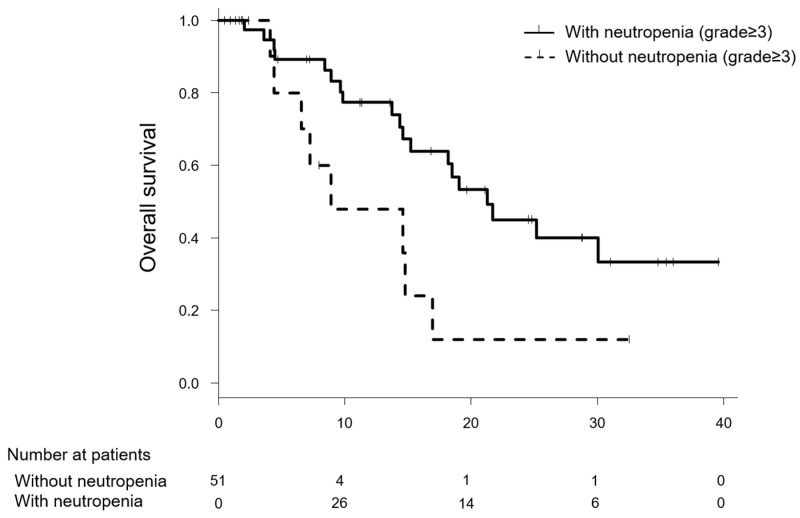
The Simon and Makuch’s modified Kaplan-Meier curves for comparison of overall survival in pancreatic cancer patients who received modified FOLFIRINOX therapy, one curve (**solid line**) represents patients with neutropenia on any study day, and the other curve (**dashed line**) represents those without grade ≥3 neutropenia on each day.

Following treatment with modified FOLFIRINOX therapy, median OS was significantly longer in patients with severe neutropenia than in those without it (21.3 months [95% CI: 15.2–NA] versus 8.9 months [95% CI: 6.6–NA]; *p* = 0.020, NA indicates calculation impossible (Figure 1). 

With time varying Cox proportional hazards regression (Table 2), the relationship between neutropenia and OS was significant (hazard ratio [HR]: 0.40; [95% CI: 0.17–0.95], *p* = 0.039) after adjusting for age and neutrophil-lymphocyte ratio (NLR). The regression model was internally validated and estimated optimism was 0.19, indicating that there was no evidence of overfitting. 

**Figure 2:**
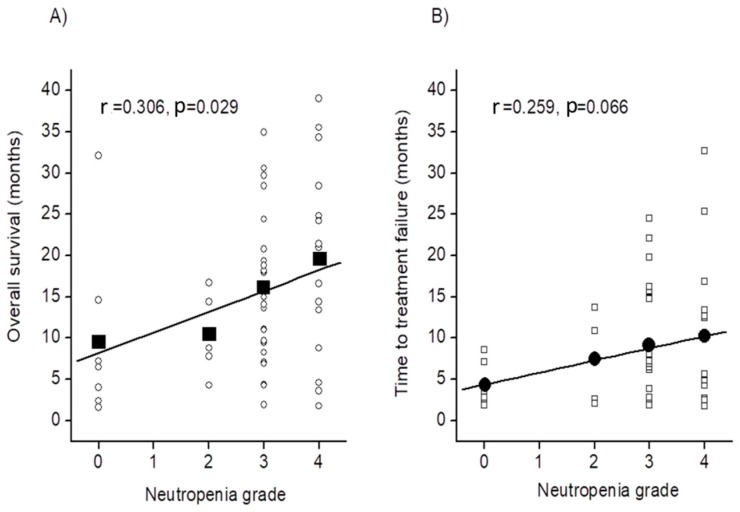
Relationship between the grade of neutropenia and overall survival (**A**) or time to treatment failure (**B**) in patients with advanced pancreatic cancer who received modified FOLFIRINOX therapy.

The median time to treatment failure (TTF) also tended to be longer in patients with severe neutropenia than in those without it (7.0 months [95% CI: 1.9–24.5] versus 3.7 months [95% CI: 2.0–12.1]; HR: 0.49 [0.22–1.09], *p* = 0.079, Table 3). Interestingly, there was a significant correlation between the grade of neutropenia and OS (r = 0.306, *p* = 0.029; Figure 2A), and TTF tended to be correlated with the grade of neutropenia (r = 0.259, *p* = 0.066; Figure 2B).

One-year survival was also slightly and not significantly higher in patients with neutropenia than in those without it [71.8% versus 41.7%; OR: 3.56 (95% CI: 0.93–13.65), *p* = 0.085]. There were no significant differences in the tumor response rates between the two groups [35.9% versus 16.7%, *p* = 0.296 for ORR; 76.9% versus 66.7%, *p* = 0.474 for disease control rate (DCR)] (Table 3).

### 2.3. Relationship between Other Factor and Prolonging Survival

To assess the relationship between candidate of prognostic factors and OS, multivariable Cox proportional hazard regression analyses were performed. With or without distant metastasis, CRP, CA19-9, NLR and modified glasgow prognostic score (mGPS) were treated as candidate of prognostic factors, and CRP, CA19-9, NLR and mGPS were treated as continuous variables. Multivariable analysis indicated that NLR was significantly associated with prolonged OS in patients with advanced pancreatic cancer who received modified FOLFIRINOX therapy (Table 4).

### 2.4. Risk Factors for Severe Neutropenia

To assess the relationship between time to neutropenia (grade ≥3) and factors which patient had at baseline, multivariable Cox proportional hazard regression analyses were performed. Neutrophil and total bilirubin were treated as continuous variables. Among these factors, Cox proportional hazard analysis showed that total bilirubin (hazard ratio [HR]: 2.65, 95% CI: 1.13–6.19, *p* = 0.024) was a significant risk factor for severe neutropenia (Table 5).

### 2.5. Comparison of the Incidence of Other Adverse Events between Patients with and without Severe Neutropenia

No significant differences in the incidence rates of such adverse events as nausea (grade ≥2), vomiting (grade ≥1), oral mucositis (grade ≥2), dysgeusia (grade ≥2), peripheral neuropathy (grade ≥2), diarrhea (grade ≥2), and thrombocytopenia (grade ≥2) were observed between patients with and without severe neutropenia (Table 6).

## 3. Discussion

FOLFIRINOX or modified FOLFIRINOX is the first-line chemotherapy for advanced pancreatic cancer, however, these regimens cause a number of severe adverse events, including neutropenia. The present study showed that a large portion of patients (76.5%, 39/51 patients) experienced grade 3-4 neutropenia during the course of modified FOLFIRINOX therapy. The occurrence of severe adverse events may cause therapy interruption or a reduction in the dose of chemotherapy drugs, thereby affecting the clinical outcomes. In the present study, the incidence of severe neutropenia led to dose reduction. In particular, the RDIs for irinotecan, oxaliplatin and 5-FU were all significantly lower in patients with severe neutropenia than in those with grades 0–2 neutropenia.

Nevertheless, it was noteworthy that the patients with grade 3–4 neutropenia showed significantly longer survival than those without severe neutropenia in the Simon and Makuch’s modified Kaplan-Meier curves, and the relationship between neutropenia and OS was significant after adjusting for age and NLR with time varying Cox proportional hazards regression. 

Additionally, severe neutropenia was a significant factor for better survival, and the OS well correlated with the grade of neutropenia. Moreover, there was also a trend towards a correlation between the grade of neutropenia and TTF. Other clinical responses, including OS rate at one-year, ORR, and DCR, also tended to be better in patients with severe neutropenia than in those without it. Therefore, severe neutropenia may be a surrogate marker for better survival outcome due to modified FOLFIRINOX therapy in patients with advanced pancreatic cancer. Our data were generally consistent with a previous report that showed that patients with severe neutropenia after treatment with gemcitabine-containing chemotherapy for advanced pancreatic cancer had significantly longer OS than those without it [19]. The survival effect of gemcitabine monotherapy for metastatic pancreatic cancer is better for patients with grade 3 neutropenia than those with lower grades of neutropenia [20].

A question that the present findings raises is: why dose the incidence of severe neutropenia yielded better survival in advanced pancreatic cancer patients receiving modified FOLFIRINOX therapy? One mechanism underlying this finding might be suppression of neutrophils themselves because these cells play a critical role in growth of tumors by promoting the acceleration of angiogenesis and suppressing the antitumor immune response [21,22]. An increase in neutrophils, which reflects systemic inflammation, might promote tumor progression by providing an advantageous environment for invasion and promotion of pancreatic cancer cells. Several studies have revealed that low neutrophil-to-lymphocyte ratio is a good predictor of prognosis in patients with pancreatic cancer [23,24]. Whether modified FOLFIRINOX therapy can also decrease the infiltration of neutrophils in pancreatic tumor tissue in patients with neutropenia should be examined in future studies.

We also presume that myelosuppression occurring in severe neutropenic patients contributes to improve the prognosis of the patients. Myeloid-derived suppressor cells (MDSCs), which reveal immunosuppressive in tumor microenvironment via inhibition of CD4+ T cell proliferation [25], accumulate in tumor cells and peripheral blood as the disease is progressed or in advanced stages of pancreatic cancer [26,27,28,29,30]. Reducing the number of MDSCs is a key antitumor mechanisms of some chemotherapy agents, including 5-FU [31], which prevents the accumulation of MDSCs in patients with pancreatic cancer [32]. Taken together, these evidence suggest that the suppressive effect of 5-FU included in FOLFIRINOX regimen on MDSCs in tumor microenvironment is more potent in patients with severe neutropenia than in those without it, where it may act to recover the cytotoxic action of T lymphocytes and prolong survival. Unfortunately, the precise mechanism underlying the 5-FU-induced reduction in the number of MDSC is unknown. Vincent et al. [31] showed that 5-FU induces apoptotic death of MDSCs by activating caspase-3 and caspase-7, and this effect of 5-FU is more potent than that of gemcitabine. They also showed that the suppressive action of 5-FU is more potent as the expression of thymidylate synthase decreases. Therefore, 5-FU in FOLFIRINOX may contribute, at least in part, to reducing the number of MDSCs induced by FOLFIRINOX. Moreover, the reduction in MDSCs may be more potent in patients with severe neutropenia than in those without severe neutropenia due to the more severely depressed thymidylate synthase activity in patients with severe neutropenia.

We found that total bilirubin level was a significant risk factor for severe neutropenia. This suggests that the biological activity of cytotoxic drugs, including irinotecan, is increased in patients with severe neutropenia because elevated bilirubin has critical effects on metabolism of such drugs [33]. Moreover, patients with severe neutropenia may have a genetic predisposition for drug metabolism that is similar to clinical course features [34]. Although the RDIs for irinotecan, oxaliplatin and 5-FU were lower in patients with severe neutropenia, these doses may still exert antitumor effects. Further biological activity and/or blood concentration of cytotoxic agents may be relatively higher in severe neutropenic patients; however, future studies are needed to evaluate these hypotheses.

## 4. Materials and Methods 

### 4.1. Patients

A total of 69 patients with advanced pancreatic cancer received first-line modified FOLFIRINOX in our outpatient chemotherapy clinic during the period between January 2014 and May 2018. Among them, 18 patients were excluded from the present study because they were treated with reduced initial doses of irinotecan due to harboring a homozygous mutation in *UGT1A1* genes such as UGT1A1*28/*28, UGT1A1*6/*6 and UGT1A1*28/*6 (*n* = 2), poor performance status (≥2 according to the Eastern Cooperative Oncology Group) (*n* = 9), or limited duration of therapy less than three cycles (*n* = 7). Data from the remaining 51 patients were analyzed in the present study. Data were obtained from electronic medical record in our hospital and analyzed retrospectively.

The present study was conducted in accordance with the guideline for human studies adopted by the ethics committee of the Gifu University Graduate School of Medicine and notified by the Japanese government (Institutional review board approval No.26-156). In view of the retrospective nature of the study, the need for informed consent from subjects was not mandated.

### 4.2. Chemotherapy

Patients were treated with modified FOLFIRINOX every 2 weeks, consisting of 2 h bolus injection of oxaliplatin at 85 mg/m^2^, 2 h bolus injection of L-leucovorin at 200 mg/m^2^, 90 min bolus injection of irinotecan at 150 mg/m^2^, followed by continuous infusion of 5-FU for 46 h at 2400 mg/m^2^ without bolus 5-FU. Patients were all administered an initial regular dose of chemotherapy in the first cycle. Dose reduction was performed in the subsequent chemotherapy cycle for patients who suffered from severe adverse events, particularly grade 3–4 neutropenia. In these patients, dose escalation was not performed even if the adverse events disappeared. The criteria for dose reduction was chosen according a Japanese phase II study on modified FOLFIRINOX [6]. Briefly, treatment was delayed when one or more of the following adverse events occurred: grade 3–4 neutropenia or thrombocytopenia, febrile neutropenia, total bilirubin >3.0 mg/dL, aspartate transaminase and alanine transaminase >150 U/L, creatinine >1.5 mg/dL, grade 3–4 peripheral neuropathy, and grade 3–4 diarrhea. Treatment was restarted after recovery from the severe adverse events, but the doses of oxaliplatin, irinotecan, and 5-FU were reduced to 65 mg/m^2^, 120 mg/m^2^, and 1200 mg/m^2^, respectively.

### 4.3. Assessment of Adverse Events

Adverse events included hematological toxicities such as neutropenia, leukopenia, and thrombocytopenia; and non-hematological toxicities, including nausea, vomiting, oral mucositis, dysgeusia, peripheral neuropathy and diarrhea. The symptoms of adverse events were graded according to the Common Terminology Criteria for Adverse Events version 4.0. [35]. The incidence rates of other adverse events than neutropenia associated with modified FOLFIRINOX therapy were compared between patients with and without severe neutropenia.

### 4.4. Risk Analysis for Grade ≥3 Neutropenia

Heterozygous mutation in *UGT1A1* genes such as UGT1A1*6/*1 or UGT1A1*28/*1, low neutrophil count and high total bilirubin concentration have been reported as risk factors for severe neutropenia in advanved colorectal cancer patients who received irinotacan-based regimen [36]. Therefore, these risk factors for severe neutropenia were examined using multivariable Cox proportional hazard analysis with adjustment for age and sex. Neutrophil and total bilirubin were treated as continuous variables.

### 4.5. Efficacy of Chemotherapy

OS was used as the primary indicator of the efficacy of modified FOLFIRINOX therapy. The tumor response rates and the time to treatment failure (TTF) were used as secondary indicators of the efficacy of modified FOLFIRINOX therapy. OS was defined as the time from the start of therapy to the death. The tumor response was evaluated using patients’ computed tomography scan as complete response (CR), partial response (PR), stable disease (SD), and progressive disease (PD), according to the Response Evaluation Criteria in Solid Tumors guideline version 1.1. [37]. The overall response rate (ORR) was defined as CR plus PR, while the disease control rate (DCR) as CR+ PR+SD. TTF was defined as the time from the start of therapy to the end of the therapy.

### 4.6. Risk Analysis for Prolonging Survival

The distant metastasis [38], CRP [38,39,40], CA19-9 [39,41,42], NLR [40,43,44,45,46,47,48] and mGPS [43,49] have been reported as prognostic factors in pancreatic cancer patients. To clarify the confounding of these factors with neutropenia on prolongation of survival, multivariable Cox proportional hazard analysis with adjustment for age and sex was performed. CRP, CA19-9, NLR and mGPS were treated as continuous variables.

### 4.7. Statistical Analyses

Data were analyzed by using IBM SPSS version 22 (IBM Japan Ltd., Tokyo, Japan) and R software version 3.5.1 (www.r-project.org). *p*-values less than 0.05 were considered significant. The characteristics of patients were summarized by median with 25th and 75th percentiles for continuous variables. Frequencies and percentages were shown for categorical variables. The Simon and Makuch’s modified Kaplan-Meier survival curves [50] was used to account for time varying exposure. For primary analysis, Cox proportional hazards regression was used to evaluate the association between severe neutropenia as a time varying exposure and OS as an outcome with adjustment for covariates. Covariates were restricted to two variables to avoid overfitting, and included age and NLR on the basis of clinical judgement and previous research, owing to their expected strongly associations with the outcome and severe neutropenia. Secondary multivariable cox regression analyses were performed to assess the association between neutropenia and potential factor with adjustment for age and sex. The reliability of the regression model was internally validated via bootstrap method by measuring of overfitting quantified by optimism parameter in a calibration plot. Bootstrap validation was performed with one hundred fifty resampling. The square of the coefficient of correlation (r) was calculated as a measure of the linearity of the relationship between the two variables.

## 5. Conclusions

Our study presented the first evidence that severe neutropenia is significantly associated with better survival in patients with advanced pancreatic cancer receiving modified FOLFIRINOX therapy. Incidence of severe neutropenia may be a useful index for the survival outcome of pancreatic cancer patients receiving modified FOLFIRINOX therapy. 

## Figures and Tables

**Table 1:** Patient demographics.

Number of patients (male/female)	51	(30/21)
Age, median (mini–max)	64	(49–74)
Heterozygous for UGT1A1*6 or *28 (with/without)	37.3	(19/32)
Height (cm)	162	(156.0–166.5)
Body weight (kg)	57	(49.0–61.5)
Body surface area (m^2^)	1.58	(1.51–1.69)
Body mass index	21.3	(19.1–23.7)
Aspartate aminotransferase (IU/L)	20	(16–31)
Alanine aminotransferase (IU/L)	19	(13–34)
Serum creatinine (mg/dL)	0.69	(0.53–0.82)
Total bilirubin (mg/dL)	0.7	(0.5–0.8)
Neutrophil (/μL)	3560	(2955–4455)
White blood cells (/μL)	5460	(4835–6345)
Hemoglobin (g/dL)	12.2	(11.5–13.1)
Platelet (10^4^/μL)	19.7	(16.1–24.6)
HbA1c (%)	6.2	(5.7–6.8)
C-reactive protein (CRP, mg/dL)	0.29	(0.05–1.42)
Carbohydrate antigen 19-9(CA19-9, U/mL)	578.9	(141.5–3983.7)
Biliary stent or drainage (%) (with/without)	29.4	(15/36)
Distant metastasis (%) (presence/absence)	54.9	(28/23)
Neutrophil-lymphocyte ratio (NLR)	2.89	(2.22–3.85)
Modified Glasgow prognostic score (mGPS, 0/1/2)	(33/10/7)	
Initial dose of chemotherapy drug		
Irinotecan (mg/m^2^)	150	
Oxaliplatin (mg/m^2^)	85	
5-Fluorouracil (mg/m^2^)	2400	

All data indicate median, 25–75th percentiles unless otherwise indicated.

**Table 2:** Cox proportional hazard analysis of time varying neutropenia associated with overall survival in advanced pancreatic cancer patients receiving modified FOLFIRINOX therapy.

Factors	Hazard Ratio (95% CI)		*p*-value
Neutropenia	0.40	(0.17–0.95)	0.039
Age (IQR:59–68)	0.90	(0.48–1.69)	0.738
NLR (IQR:2.2–3.8)	1.25	(1.02–1.52)	0.029

Cox proportional hazards regression with neutropenia as a time-varying exposure, with adjustment for age and NLR. Abbreviations: IQR, inter quartile range; CI, confidence interval; NLR, neutrophil-lymphocyte ratio.

**Table 3:** Comparison of the median time to treatment failure and tumor response rate between patients with grade ≥3 neutropenia and those without it after treatment with modified FOLFIRINOX for advanced pancreatic cancer.

Effect	Without Neutropenia (*n* = 12)		With Neutropenia (*n* = 39)		*p*-value
Median time to treatment failure (months, 95% CI)	3.7	(2.0-12.1)	7.0	(1.9-24.5)	0.079
Tumor response rate (%)					
Response rate (CR+PR)	16.7	(2/12)	35.9	(14/39)	0.296
Disease control rate (CR+PR+SD)	66.7	(8/12)	76.9	(30/39)	0.474
One-year survival (%)	41.7	(5/12)	71.8	(28/39)	0.085

Data were statistically analyzed by Fisher’s exact probability test. CI: confidence interval CR: complete response; PR: partial response; SD: stable disease

**Table 4:** Cox proportional hazard analysis of the risk factors associated with overall survival among various adverse events observed in advanced pancreatic cancer patients receiving modified FOLFIRINOX therapy.

Factors	Univariable Analysis			Multivariable Analysis		
	HR	(95% CI)	*p*-value	HR	(95% CI)	*p*-value
With distant metastasis	2.07	(0.83–5.16)	0.119	2.11	(0.84–5.30)	0.113
CRP	1.01	(0.84–1.22)	0.908	0.99	(0.82–1.21)	0.953
CA19-9	1.00	(0.99–1.00)	0.122	1.00	(0.99–1.00)	0.181
NLR	1.18	(1.02–1.36)	0.030	1.15	(1.00–1.34)	0.048
mGPS	0.90	(0.55–1.47)	0.676	0.92	(0.56–1.53)	0.755

Hazard ratio (HR) and 95% confidence intervals (CI) were indicated. All multivariable analyses were performed adjusting for age and sex.

**Table 5:** Cox proportional hazard analysis of the risk of grade≥3 neutropenia in pancreatic cancer patients receiving modified FOLFIRINOX therapy.

Factors	Univariable Analysis		Multivariable Analysis	
	HR (95% CI)	*p*-value	HR (95% CI)	*p*-value
Heterozygous for UGT1A1*6 or *28	1.41 (0.74–2.70)	0.300	1.35 (0.69–2.65)	0.373
Neutrophil	1.00 (0.99–1.00)	0.062	1.00 (0.99–1.00)	0.074
Total bilirubin	2.79 (1.30–6.10)	0.010	2.65 (1.13–6.19)	0.024

Hazard ratio (HR) and 95% confidence intervals (CI) are indicated. All multivariable analyses were performed adjusting for age and sex.

**Table 6:** Comparison of the incidence of other adverse events (grade ≥2) between patients with and without grade ≥3 neutropenia.

Adverse Effect	Without Neutropenia (*n* = 12)		With Neutropenia (*n* = 39)		*p*-value
	%	(presence/absence)	%	(presence/absence)	
Nausea	58.3	(7/5)	53.8	(21/18)	1.000
Vomiting	8.3	(1/11)	10.3	(4/35)	1.000
Oral mucositis	16.7	(2/10)	20.5	(8/31)	1.000
Dysgeusia	8.3	(1/11)	25.6	(10/29)	0.422
Peripheral neuropathy	41.7	(5/7)	28.2	(11/28)	0.481
Diarrhea	33.3	(4/8)	25.6	(10/29)	0.715
Leukopenia	33.3	(4/8)	100	(39/0)	<0.001
Thrombocytopenia	16.7	(2/10)	25.6	(10 / 29)	0.706

Data were statistically analyzed by Fisher’s exact probability test.
